# Habitat Protection Indexes - new monitoring measures for the conservation of coastal and marine habitats

**DOI:** 10.1038/s41597-022-01296-4

**Published:** 2022-05-12

**Authors:** Joy A. Kumagai, Fabio Favoretto, Sara Pruckner, Alex D. Rogers, Lauren V. Weatherdon, Octavio Aburto-Oropeza, Aidin Niamir

**Affiliations:** 1 Senckenberg Biodiversity and Climate Research Center, Frankfurt am Main, Germany; 2 Centro para la Biodiversidad Marina y Conservación, A.C., La Paz, Baja California Sur Mexico; 3 Universidad Autónoma de Baja California Sur, La Paz, Baja California Sur Mexico; 4 UN Environment Programme World Conservation Monitoring Centre (UNEP-WCMC), Cambridge, United Kingdom; 5 REV Ocean, Lysaker, Norway; 6 Scripps Institution of Oceanography, University of California San Diego, La Jolla, California USA

**Keywords:** Marine biology, Conservation biology

## Abstract

A worldwide call to implement habitat protection aims to halt biodiversity loss. We constructed an open-source, standardized, and reproducible workflow that calculates two indexes to monitor the extent of coastal and marine habitats within protected areas and other effective area-based conservation measures. The Local Proportion of Habitats Protected Index (LPHPI) pinpoints the jurisdictions with the greatest opportunity to expand their protected or conserved areas, while the Global Proportion of Habitats Protected Index (GPHPI) showcases which jurisdictions contribute the most area to the protection of these habitats globally. We also evaluated which jurisdictions have the highest opportunity to contribute globally to protecting habitats by meeting a target of 30% coverage. We found that Areas Beyond National Jurisdiction (ABNJ) have the greatest potential to do so. Our workflow can also be easily extended to terrestrial and freshwater habitats. These indexes are helpful to monitor aspects of the Sustainable Development Goal 14 and the emerging post-2020 global biodiversity framework, to understand the current status of international cooperation on coastal and marine habitats conservation.

## Introduction

Coastal and marine habitats face simultaneous global and local pressures, with more than half of the ocean experiencing increasing cumulative impacts in the last decade^1^. These habitats provide critical services to humans (i.e., nature’s contributions to people), spanning regulating (e.g., coastal protection and CO_2_ sequestration), provisioning (e.g., food), supporting (e.g., habitat provision), and cultural services (e.g., science and well-being). Yet habitat destruction has continued into the 21st century, with the loss of one-third to one-half of vulnerable coastal and marine habitats globally, accompanied by a reduction in nature’s contributions to people^2^. To help solve this worldwide crisis, there is a clear call for the use of data to drive decision-making. Various organizations have made this call, including the High-Level Panel for a Sustainable Ocean Economy^3^, the High Ambition Coalition for Nature and People, and the Intergovernmental Oceanographic Commission (IOC) through the UN Decade of Ocean Science for Sustainable Development^4^.

Protected or conserved areas (PCAs)^5^ are critical to maintaining the health of ecosystems and their contributions to people. When protected areas are adequately planned, designated, implemented, and managed^6,7^, habitat integrity is secured, depleted fish populations can rebound, and biodiversity can increase, leading to positive spillover effects^8,9^. Regulation and correct placement of protected areas are fundamental to safeguarding ecosystems effectively. Although undesirable outcomes can result from ineffective implementation of marine protected areas^10–12^, it has been shown that 90% of the maximum potential biodiversity benefits from marine protected areas can be achieved by strategically protecting 21% of the ocean (43% of exclusive economic zones and 6% of the high seas)^13^. Defined in 2018 by the Convention on Biological Diversity^14^, other effective area-based conservation measures (OECM) are an additional tool that can advance equitable and effective conservation. OECMs can enhance the recognition of the areas managed by Indigenous and local communities that sustain local biodiversity and can help to ensure a well-connected conversation network^15^. Together, PCAs have the opportunity to be a suite of effective and equitable tools to enhance biodiversity in varied contexts worldwide.

As of January 2022, 7.92% of the global ocean area was covered by marine protected areas^16,17^, and 2.8% was within fully protected areas (https://mpatlas.org/). Target 2 of the zero draft of the post-2020 global biodiversity framework currently under negotiation proposes that by 2030 at least 30% of the earth’s surface should be protected or conserved through a well-connected and effective system of protected areas and other effective area-based conservation measures with the focus on areas particularly important for biodiversity^18,19^. Whether or not this is agreed upon, setting targets for ecosystem conservation is essential for policy-makers^20^.

However, international targets can be achieved in terms of area while failing the overall strategic goal because the areas are poorly located. To prevent ‘paper-parks,’ PCAs should be effectively managed and ecological representative to deliver the highest biodiversity benefits. Only 11% of protected areas, representing 18.29% of global protected area coverage, have been assessed for their management effectiveness, and only 15.4% of countries meet their targets of assessing at least 60% of protected areas for their effectiveness^16^. By December 2020, just 9% of marine protected areas had an associated management effectiveness assessment published within the Global Database of Protected Area Management Effectiveness^21^. To deliver the maximum potential biodiversity benefits, PCAs also need to include a sufficient representation of the world’s habitats and species^16,22^. Aichi target 11 highlights this by mentioning “ecologically representative” protected areas. So far, the increase in ecological representation has been a byproduct of covering more area as opposed to carefully considering ecological representation^23^. By providing performance metrics, society can monitor that achieving the required area in the targets is not simply the end but generates genuine benefits for biodiversity^20^.

Credible metrics are critical to evaluate whether expanding protected areas achieve conservation outcomes and objectives. For metrics to be valid and easily adopted, they must be transparent and easy to understand. There have been previous efforts to quantify the ecological representation of protected areas, such as the protection equity metric^24,25^ and the mean protection gap metric^26^. These are valuable tools to pinpoint how some bioregions are misrepresented within a network of protected areas and progress from previous metrics that emphasize the total area protected^24–26^.

Our indexes complement these existing tools measuring progress towards better representation in area-based targets: the Global Proportion of Habitats Protected index (GPHPI), which is the area of habitats within PCAs in a jurisdiction divided by the global extent; and the Local Proportion of Habitats Protected Index (LPHPI) defined as the average area of habitats within PCAs in a jurisdiction divided by the extent of the habitats in the same jurisdiction. Together the indexes inform countries about ecological representativeness and the amount of protection within each jurisdiction worldwide. Our indexes and workflow can be distinguished from previously published metrics in four critical aspects:

i) The indexes follow the FAIR (Findable, Accessible, Interoperable, Re-usable) principles as the workflow guides the user in every step from the data preparation to the index calculation, as opposed to similar indexes (e.g., mean protection gap metric^26^) that rely on a localized case by case application and do not provide tools for data preparation, which often hinders the practical application of the indexes. In this way, our approach helps standardize the entire workflow to better compare the amount of marine and coastal habitats within PCAs both among local areas (LPHPI) and at a global level (GPHPI).
ii) The indexes are spatially explicit and present a global context for the conservation of habitats, through showing both the necessity of corporation and individual jurisdictions’ potential to contribute to conservation efforts.
iii) The indexes can easily be compared to a target but are independent of it. Furthermore, the indexes are flexible and are easy to update as new data with similar or larger resolutions become available.
iv) The indexes apply equally to areas beyond national jurisdiction and can be extended to terrestrial and freshwater habitats.

These indexes, along with the workflow provided, shed light on the global effort governments are putting towards marine and coastal habitat conservation and can be used for global-level policy and national statistical offices. The output and interpretation of these indexes are particularly relevant to guide future international agreements as countries should enhance coordination to meet multiple objectives and prioritize conservation outcomes over individual targets^23–25^.

## Results

**Fig. 1:**
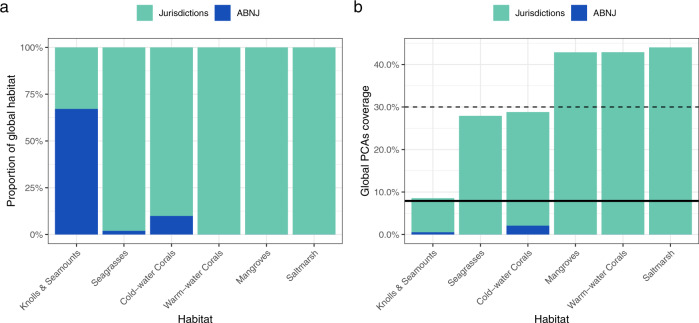
Overview of the distribution and protection of the six habitats globally separated by location within jurisdictions or ABNJ. (**a**) Illustrates the extent distribution of each habitat between jurisdictions and ABNJ. Warm-water corals, mangroves, and saltmarsh do not occur in ABNJ; (**b**) Represents the global coverage of PCAs for each habitat divided between jurisdictions and ABNJ. The dashed line at 30% represents the target that 30% of the ocean should be conserved by 2030, while the solid black line at 7.74% represents the current protection of the ocean surface area according to UNEP-WCMC^16^.

On a global level, more than 42% of mapped warm-water corals, mangroves, and saltmarshes fall under PCAs, while 27–29% of mapped seagrasses and cold-water corals are under PCAs (Fig. 1). Knolls and seamounts have the least area covered by PCAs, with a total of 8.5%. If we consider just ABNJ, where 67% of the extent of knolls and seamounts are located (Fig. 1a), only 0.8% are under PCAs. The discrepancy between the protection of coastal regions and ABNJ results from an overall focus on national waters and the underlying challenges faced by international conservation efforts within ABNJ. Since half of the selected habitats are found within ABNJ (cold-water corals, seagrasses, and knolls and seamounts), it is important to implement new measures to protect these areas.

**Fig. 2:**
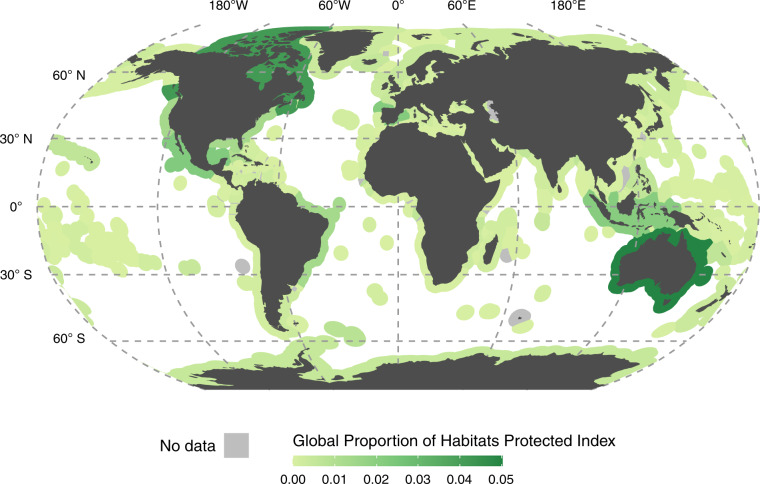
The Global Proportion of Habitats Protected index (GPHPI). GPHPI illustrates the contribution of jurisdictions to the global coverage of six marine and coastal habitats by PCAs, ranging from yellow-green (low contribution) to dark green (high contribution). The GPHPI is calculated by taking the average of each habitat specific GPHPI presented in Supplementary Information 1. Habitats that do not occur in the jurisdiction’s extent are not included in the calculation. The distribution of the index is highly right-skewed, with a few jurisdictions with comparatively very high scores and many jurisdictions with low scores. The ABNJ index value is not depicted for clarity but is 0.0087. The GPHPI hypothetically ranges from 0 to 1, but only 0 to 0.05 is shown here due to no jurisdictions scoring higher than 0.05.

At the jurisdiction level, we selected 242 jurisdictions that include at least one of the habitats used in the analysis. The GPHPI measures how much a jurisdiction contributes to the total extent of the marine and coastal habitats we consider within PCAs globally. The top five jurisdictions with the highest average GPHPI value are Australia, Canada, Mexico, Spain, and Indonesia (Fig. 2), which are within the top 10 jurisdictions with the largest economic exclusive zone area except for Spain. Notably, the GPHPI has a highly right-skewed distribution, meaning that just a few jurisdictions contribute extensively to habitat protection on a global scale. These top five jurisdictions together contribute 15.8% (GPHPI score of 0.158) of the average global protection of the considered marine and coastal habitats by extent.

**Fig. 3:**
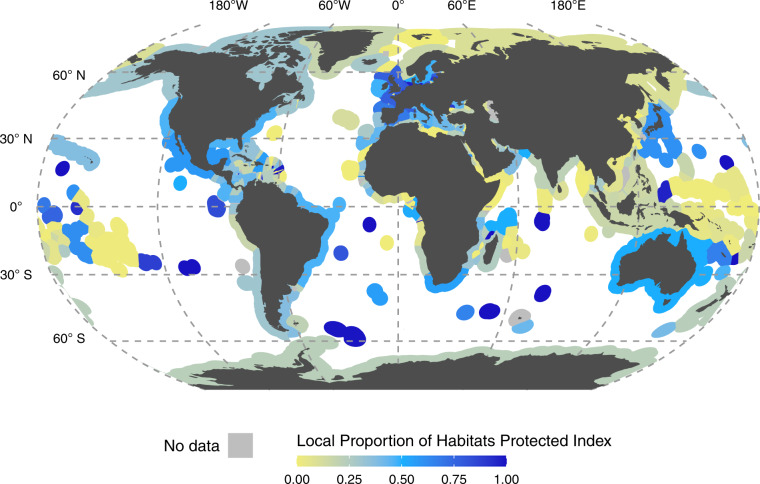
The Local Proportion of Habitat Protected Index (LPHPI). LPHPI illustrates how much a jurisdiction covers the six marine and coastal habitats considered with PCAs compared to the maximum habitat extent, ranging from yellow (low contribution) to dark blue (high contribution). The index ranges from 0 to 1, indicating no coverage to 100% coverage of PCAs. The LPHPI is calculated by taking the average of each habitat specific LPHPI presented in Supplementary Information 1. Habitats that do not occur in the jurisdiction’s extent are not included in the calculation. The ABNJ index value is not depicted for clarity but is 0.072.

Comparatively, the LPHPI, which measures how much coastal and marine habitat in a jurisdiction is within a PCA on average, also has a right-skewed distribution. The LPHPI varies greatly over the jurisdictions (Fig. 3) and for the six selected marine and coastal habitats when calculated individually (Supplementary Information 1). More than half of jurisdictions have an LPHPI value of less than 0.27, and 26% of jurisdictions (63) have an LPHPI value higher than 0.50. Four jurisdictions have an LPHPI value of 1, indicating that all their mapped habitats we consider occur entirely within PCAs. In comparison, 28 jurisdictions have a value of 0, where none of these habitats are within PCAs. Many of the top LPHPI values are held by island jurisdictions. For example, Bouvet Island, Sint-Eustatius, and Serrana Bank all have a LPHPI value of 1.

**Fig. 4:**
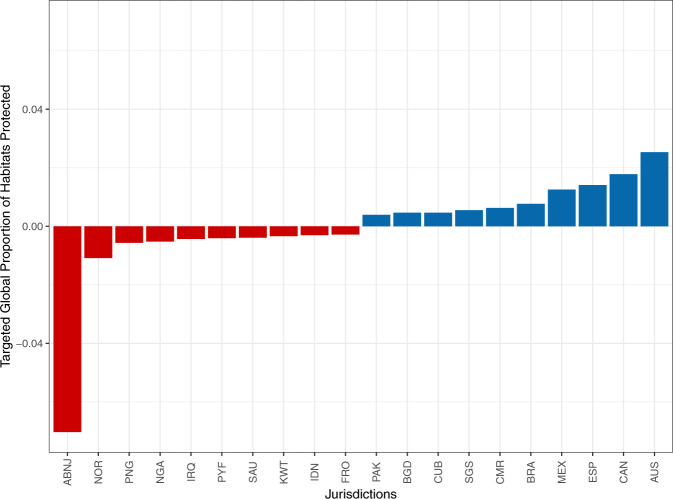
Results from the targeted analysis of the global proportion of habitats protected. The top 10 and bottom 10 out of 242 jurisdictions ranked according to the results from the targeted analysis, illustrating whether these countries have on average 30% of their habitats within PCAs. Jurisdictions in blue (above 0) have on average more than 30% of their habitats’ extent within PCAs, while jurisdictions in red (below 0) have on average less than 30% of their habitats’ extent within PCAs. Jurisdiction names correspond to ISO3 names except for the ABNJ.

Finally, an analysis was conducted to calculate an additional benchmark that measures how far away jurisdictions are from protecting 30% of their habitats (Fig. 4; Supplementary Information 2 for habitat specific figures). The targeted analysis of the global proportion of habitats protected subtracts 30% of each habitat from the GPHPI (see methods) so that jurisdictions with positive values (n = 119) on average have more than 30% of the selected habitats within PCAs. In contrast, jurisdictions with negative values (n = 123) are not meeting this goal. The top five jurisdictions with positive values in the targeted analysis are Australia, Canada, Spain, Mexico, and Brazil. Interestingly, ABNJ rank the lowest in this analysis even though they rank 10th highest on the GPHPI. Three of the six habitats considered fall within ABNJ. However, only a small proportion of ABNJ are within PCAs, leading to this disconnect. The index values for the LPHPI, GPHPI, and the results of the targeted analysis can be found in the accompanying repository.

## Discussion

There are 23 international conventions related to protecting the marine environment and biodiversity, with five of these requiring the implementation of marine protected areas^27^. Targets for the effective protection of marine habitats that conserve nature and secure nature’s contributions to people are increasingly seen as critical in ensuring progress toward meeting treaty commitments. Aichi Target 11 and the Sustainable Development Goals Target 14.5 aim to conserve at least 10% of marine and coastal areas by 2020, reflecting a shift to a more target-driven conservation policy at the international level, although this is hotly debated. Warm-water corals, mangroves, and saltmarshes all have more than 30% of their extent within PCAs, with seagrasses and cold-water corals approaching 30%, which reveals a dedicated effort to their conservation of these critical habitats. However, the protection of the total global ocean area is still at 7.92%, with only 1.18% of ABNJ covered by PCAs, falling short of the 10% of Aichi Target 11 previously set for 2020^16^.

Standardized and open source tools and platforms are needed to allow robust monitoring of progress towards international targets. While tools are available to measure advancement in some targets^24–26^, fully replicable workflows that guide the user from data preparation to index calculations have been lacking. The workflow presented here provides one of the first steps to fill this gap. The indexes also give a global context for the conservation of the habitats, highlighting ecological representation and individual jurisdictions’ potential to contribute to future conservation efforts. Combining our indexes with other tools, such as spatial conservation planning, allows policymakers to balance tradeoffs with different priorities, such as climate mitigation and resource extraction (e.g.^13^).

While our indexes do not measure the “equitably managed” component of the Aichi target 11, it is critical that a holistic, human rights-based approach is taken in meeting any targets set and efforts to improve biodiversity outcomes. The consideration of human rights of local communities and indigenous people and inclusion of their voices is absolutely necessary in the decision-making process^28^.

### Interpretation and Usefulness of the Workflow and Indexes

**Fig. 5:**
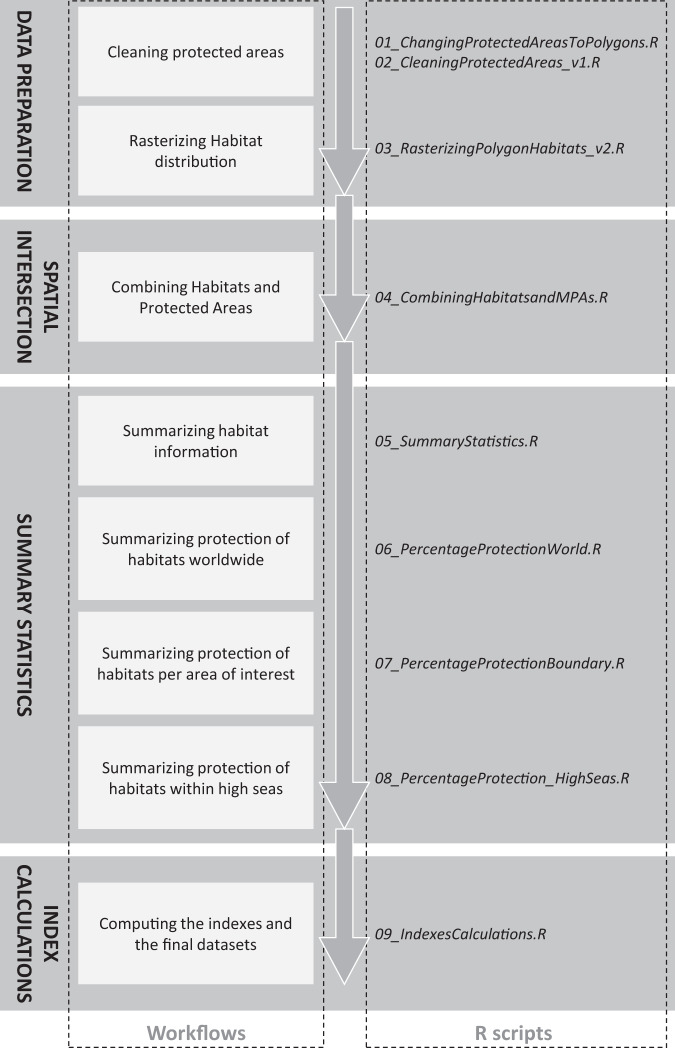
A flow chart describing the key steps of the indexes calculations. We also connect each step to the R script available at: https://github.com/jkumagai96/Marine_Habitat_protection where a more detailed explanation on how to replicate the workflow is available.

The LPHPI and GPHPI are consistent ways of measuring progress in establishing protected areas that have the potential to conserve habitats and biodiversity. Additionally, the completely open access workflow described in Fig. 5 is highly adaptable and can include a wide range of habitats as data become available, or it can be applied to different conservation features like species distributions. The workflow could also be adapted to calculate the amount of key biodiversity areas within PCAs per jurisdiction and globally, or human threats (e.g., pollution or heatwaves) when geospatial data is available. Notably, the workflow can also measure progress towards targets in the draft post-2020 global biodiversity framework (as of August 2020).

Specifically, the workflow and resulting LPHPI dataset can directly monitor the marine components T2.1 and T2.3 of Target 2 of the draft monitoring framework (reproduced in Table 1 for convenience). The workflow can also be easily adapted to calculate the freshwater and terrestrial aspects of Target 2 – component T2.1 and component T2.2. The Protected Area Representativeness Index and Species Protection Index currently proposed for T2.3 do not account for marine regions or species. We provide more data directly on the other indicator mentioned (Proportion of terrestrial, freshwater, and marine ecological areas within PCAs) for marine areas in a FAIR workflow. Our workflow and indexes are useful resources that monitor Target 2 of the draft monitoring framework for the post-2020 global biodiversity framework. Additionally, the inclusion of ABNJ in the indexes is extremely important given current discussions on a new implementing agreement for the United Nations Convention on the Law of the Sea to protect marine biodiversity in areas beyond national jurisdiction and thus the whole ocean^29^.

**Table 1:** Subset of the draft monitoring framework for the post-2020 global biodiversity framework available online (https://www.cbd.int/sbstta/sbstta-24/post2020-monitoring-en.pdf).

Updated 2030 Targets	Components of the 2030 Targets	Monitoring Elements	Indicators
**Target 2** By 2030, protect and conserve through well connected and effective system of protected areas and other effective area-based conservation measures at least 30% of the planet with the focus on areas particularly important for biodiversity	**T2.1** Area of terrestrial, freshwater and marine ecosystem under protection and conservation	Trends in extent of protected areas	Protected area coverage.
			Coverage of protected areas in relation to marine areas (SDG indicator 14.5.1)
			Coverage by protected areas of important sites for mountain biodiversity (SDG indicator 15.4.1)
		Trends in extent of areas under other area-based conservation measures	Coverage of other effective area-based conservation measures
	**T2.2**. Areas of particular importance for biodiversity are protected and conserved as priority	Trends in proportion of areas of particular importance for biodiversity protected and conserved	Protected Area Coverage of key biodiversity areas
			Proportion of important sites for terrestrial and freshwater biodiversity that are covered by protected areas, by ecosystem type
			Species Protection Index
			Proportion of important sites for terrestrial and freshwater biodiversity that are covered by protected areas, by ecosystem type (SDG indicator 15.1.2)
	**T2.3**. Representative system of protected areas and other effective area-based conservation measures	Trends in ecological representativeness of areas conserved	Protected Area Representativeness Index (PARC-Representativeness)
			Proportion of terrestrial, freshwater and marine ecological regions which are conserved by PAs or OECMs.
			Species Protection Index.

The GPHPI is a valuable index that reveals the protection status of habitats distributed globally. The index highlights that not all countries have the same amount of habitat, and international effort is needed to conserve biodiversity worldwide, aspects that the LPHPI does not readily show. It is valuable to understand where habitats are covered by protected areas and where further efforts need to be placed. For example, Norway has a relatively low LPHPI (0.168) and simultaneously a relatively high GPHPI (top 11%) because of the total area of mapped habitats within their jurisdiction and their efforts to conserve them. If they can improve their LPHPI to 0.3 (30%), their GPHPI would also increase since they have a large area of habitats. But even with less than 30% of these habitats in PCAs, the protection Norway has established, or other countries have in a similar situation, substantially contributes to the global effort.

Jurisdictions have direct control over their LPHPI. Increasing the protected area coverage of their marine and coastal habitats will directly increase the index score. Small countries and territories with a limited area may see large improvements in their LPHPI through a few additional protected areas, while their GPHPI score will not increase much from this effort. For these jurisdictions, international strategies need to be implemented to promote the conservation of marine and coastal habitats. The GPHPI also reveals that each jurisdiction may physically contribute only a small percentage. However, when combined, these could provide the overall coverage of PCAs distributed around the world that is ecologically advisable to promote overall biodiversity.

Within the targeted analysis of the global proportion of habitats protected, any jurisdiction that protects more than 30% of its habitat extent can move from a negative to a positive score; thus, it is relative to each jurisdiction. However, the targeted analysis also reflects the absolute contribution of each jurisdiction. In particular, the targeted analysis can be interpreted to reveal jurisdictions that have the highest opportunity to conserve the most habitat, if they can reach the 30% target. Thus, this informs part of goal D of the post-2020 biodiversity framework, which requires understanding where to prioritize effort. The jurisdictions that rank the lowest in the analysis, currently ABNJ, Norway, Papua New Guinea, Nigeria, and Iraq (Fig. 4), represent a great opportunity to further expand PCAs to 30% coverage of marine habitats within their territorial waters and coast, as these would contribute the most added area. The jurisdictions that score highest have the opportunity to monitor and improve the effectiveness of their PCAs to adequately protect these marine habitats and reduce surrounding pressures, especially since they contribute significantly to the total global extent of these habitats.

### Limitations

One limitation of our indexes is that they do not distinguish between areas that are readily protected (e.g., due to remoteness) and those that most urgently need protection (e.g., highly threatened biodiverse locations)^30,31^. Additionally, the analysis presented here is sensitive to the choice of coastal and marine habitats included in the indexes. We selected these six habitats based on the availability of high-quality spatially explicit global data recognized by the scientific community. Each habitat dataset is published in a peer-reviewed journal and available online (https://data.unep-wcmc.org/datasets) within the UN Environment Programme World Conservation Monitoring Centre (UNEP-WCMC) website and follows their data standards. The data represent the known and mapped distribution of habitats; thus, there are inherent knowledge gaps between the actual extent and available data. For example, it is likely that significant portions of cold-water corals, particularly in the ABNJ, are still unknown. Over time, the workflow will be updated and improved yearly to strengthen data coverage, and if additional high-quality data on habitats emerge, these will be included ensuring the indexes stay up to date and relevant. The original analysis with the same habitats will also be repeated to ensure a consistent time series of the indexes is provided.

An important consideration when using these indexes is that habitat extent that spatially aligns with a PCA does not necessarily mean that a particular habitat is protected. For example, some PCAs enforce regulations on the water area (e.g., fishing exclusion), but do not prevent mangrove deforestation. Additionally, because of the buffering of points within the workflow, some of the habitats that are counted as protected may fall near a PCA but not within it. Nevertheless, our analysis assumes that habitats that fall within a PCA will be better conserved than habitats not within a PCA, as the primary purpose of protected areas is conservation. Similarly, we assume that other effective area-based conservation measures provide some conservation benefit and are often sustainably managed by local communities and indigenous peoples who live on them^32,33^.

The LPHPI and GPHPI indexes report detailed information for policymakers, the scientific community, and stakeholders to understand the state of protection for marine and coastal habitats at both global and local levels. Simple metrics like these indexes that the public and politicians understand help communicate the plight of ocean health and efforts to improve it. The workflow, based on open-source programming and datasets, is reproducible and scalable and was developed to allow other scientists and data providers to calculate the indexes for any areas or habitats of interest and repeat and adapt our analysis for any target. The indexes will be updated annually to ensure continued relevance and the provision of a time series to track how the world is advancing towards the goals defined by global policy, such as aspects of the Sustainable Development Goal 14, therefore bringing to the forefront the importance and status of conserving critical marine and coastal habitats. Ultimately, transparency in protection efforts, effectiveness, and representation must be improved so policymakers can grasp the current conditions, possible scenarios, and make informed decisions to meet international policy commitments^34^.

## Methods

The two indexes presented in this paper were created using the free and open-source programming language, R, and open-access datasets frequently used by the scientific community. We focused on six marine and coastal habitats and analyzed the protection of habitats in light of the proposed target of 30% protection. We created a standardized and reproducible workflow (Fig. 5) described below to calculate the indexes, following the FAIR data management principles. All datasets included in the workflow are listed in Table 2, and all of the code can be accessed in the Zenodo repository (10.5281/zenodo.4694821).

**Table 2:** Description of all data sources used in the workflow.

Name	Website and Reference	Date of Access	Version
World Database on Protected Areas	Available online at: https://www.protectedplanet.net/en/thematic-areas/wdpa	January 2022	January 2022
World Database on Other Effective Area-Based Conservation Measures	Available online at: https://www.protectedplanet.net/en/thematic-areas/oecms	January 2022	January 2022
Union of EEZs and countries	Available online at: https://www.marineregions.org/^40^	December 2020	Version 3
EEZs	Available online at: https://www.marineregions.org/^41^	March 2021	Version 11
Cold-water Corals	Available online at^42^	January 2022	Version 5.1
Warm-water Corals	Available online at^43^	January 2022	Version 4.1
Knolls and Seamounts	Available online at: https://data.unep-wcmc.org/datasets/41	March 2021	Version 1.0
Mangroves	Available online at^44^	December 2020	GMW 2016
Saltmarshes	Available online at^45^	January 2022	Version 6.1
Seagrasses	Available online at^46^	January 2022	Version 7.1
Ocean	Available online at: https://www.naturalearthdata.com/downloads/110m-physical-vectors/110m-ocean/	December 2020	Version 4.1.0

### Data collection and processing

To calculate the coverage of PCAs for each of the habitats, the first step of the workflow was to download, clean, and filter the World Database of Protected Areas (WDPA) (available online at https://www.protectedplanet.net/en/thematic-areas/wdpa) and the World Database on Other Effective Area-based Conservation Measures (WD-OECM)(available online at https://www.protectedplanet.net/en/thematic-areas/oecms). We followed the Protected Planet Initiative method for the protected and conserved areas described on https://www.protectedplanet.net/en/resources/calculating-protected-area-coverage but adapted it to the R programming language. We removed UNESCO Man and the Biosphere (MAB) Reserves, protected areas reported with a status of ‘Proposed’ or ‘Not Reported’, or reported as points with no reported area. Points with a reported area were buffered to create polygons matching the reported area for all PCAs. Next, the PCAs were re-projected to the Behrmann equal-area projection. All PCA layers were then converted into a raster of 1 km^2^ pixels and merged.

To develop our workflow, we selected six coastal and marine habitats from an authoritative source of internationally recognized data, UNEP-WCMC’s Ocean Data Viewer. We selected these six habitats based on their ecological importance and availability of high-quality spatially explicit global data identified by the scientific community. The Ocean Data Viewer supplies downloadable data after an approved data standard procedure before dissemination. The six habitats selected are cold-water corals, warm-water corals, knolls and seamounts, mangroves, saltmarshes, and seagrasses. All six habitats were downloaded from the Ocean Data Viewer. Importantly, the union of knolls and seamounts’ base area was calculated and used. Habitat layers were projected and converted into rasters. For points, we buffered them to create polygons with an area equaling the reported area; if the points had no area assigned, we assumed they had a 1 km^2^ extent.

The habitat rasters were then intersected with the PCAs raster. Therefore, two layers were produced for each habitat—one representing the habitat in 1 km^2^ pixels and the second representing the habitat within PCAs. We then extracted the number of pixels within each area of interest for all resulting layers. Our areas of interest were the union of countries or territories and their respective economic exclusive zones. We used the union of land and their exclusive economic zones remaining at the territory level to ensure that the coastline did not clip the extent of coastal habitats such as mangroves and saltmarsh. Landlocked countries and disputed and joint-regime areas were also filtered from the dataset; therefore, the global extent used in the indexes’ calculation is the sum of the habitat extent of the remaining jurisdictions. The global statistics reported in Fig. 1, though, were calculated based on the number of pixels within each habitat layer, independent of the areas of interest. We also calculated the number of pixels within ABNJ with the same method. While rasterizing polygon data may decrease area accuracy, due to the high-resolution grid used, 1 km^2^, minimal accuracy was lost. Comparing the current area reported for each of these habitats available from Ocean+^35^, we had less than a 0.5% difference of area from this conversion.

No-take areas and effectively managed areas can easily be added to our workflow. Considering that numerically 99.1% of the areas in the WDPA are assigned to the categories “not applicable” or “not reported” regarding their no-take status, it was decided not to include any data on no-take areas for our indexes. For example, in Mexico, their national databases have 329,875 km^2^ of the area within no-take protected areas (4.8% of the exclusive economic zone)^36,37^, yet zero no-take areas are reported in the WDPA in Mexico. Once there is more comprehensive global data on no-take statuses, these areas can be easily included as a separate category of our indexes because of their demonstrated conservation importance. Other effective area-based conservation measures are included in the index calculations as they are explicitly mentioned in Target 2 of the draft post-2020 global biodiversity framework currently being negotiated^18,19^. The current World Database on Other Effective Area-based Conservation Measures (WD-OECM)^26^ only has data reported from eight countries and territories and 671 records, but further efforts to collect data on these areas having initiated following the 14th Conference of Parties of the Convention on Biological Diversity in 2018 (Decision 14/8)^38^.

### Index calculations and considerations

Using the resulting data, we first calculated the average percent of the marine and coastal habitats falling within a PCA for each jurisdiction. Then, we divided the total number of a specific habitat’s pixels within PCAs by the total number of the same habitat’s pixels for each area of interest (Supplementary Information 1). We then averaged the resulting numbers for the six selected habitats, resulting in the Local Proportion of Habitats Protected Index (LPHPI, Fig. 3). The LPHPI measures how much a jurisdiction covers its habitat extent with PCAs, compared to their total habitat area. The Global Proportion of Habitats Protected Index (GPHPI) measures how much each jurisdiction contributes to the total extent of habitats within PCAs globally. The GPHPI is calculated by dividing the extent of each habitat within PCAs within a jurisdiction by the global extent of that habitat (sum of each jurisdiction’s habitat extent) (Supplementary Information 1). We then averaged this value for the six habitats (Fig. 2). The theoretical range for both indexes is from 0 to 1.

Finally, we argue that at least 30% of these habitats should be protected due to the high value of their contributions to people and high importance for biodiversity protection coupled with the current discussions of the post-2020 global biodiversity framework^18^. With this in mind, we conducted an analysis that evaluates how well jurisdictions are meeting this goal. Each jurisdiction’s target extent of habitat within PCAs is calculated by multiplying its global fraction of habitat by 0.3 and subtracting this from the GPHPI (habitat-specific). This calculation is done for each habitat (Supplementary Information 2, Figures 7–12), and the average of all habitats is presented in Fig. 4.

The workflow runs at a 1 km^2^ scale; thus, small patches of habitat polygons that do not intersect the centroid of each raster cell will not be included in the rasterization process. If the habitat polygon intersects with the centroid, it will be rasterized to a 1 km^2^ resolution. Additionally, the final indexes are the average protection for all habitats present in the jurisdiction being considered. Some areas or countries with very little habitat in small patches have a value of 1 for the LPHPI because the limited areas of the mapped habitat that were rasterized fall within a PCA. For example, Bouvet Island has 50 pixels of knolls and seamounts in the dataset and all fall within a protected area. No other habitats are present according to the datasets; therefore, the jurisdiction has an LPHPI value of 1. Because of this rasterization process, there may be small patches of habitat that are not under a protected or conserved area within the country or territory and are not reported here.

## Supplementary information

Supplementary Information 1

Supplementary Information 2

## Data Availability

The final dataset in CSV format is available on Zenodo (10.5281/zenodo.6325199)^39^, consisting of the LPHPI and GPHPI, and the results of the targeted analysis of the global proportion of habitats protected for each jurisdiction. An additional dataset with more detailed information is included where the indexes for each habitat per jurisdiction are reported alongside the habitat specific 30% analysis results. These indexes will be updated annually and include trends over time after a few years. Additionally, these indexes will be further improved over time with additional habitats as more detailed spatial habitat data becomes openly available on a global scale, and the World Database of Protected Areas and World Database of Other Effective Area-based Conservation Measures continues to be updated with higher resolution data and the statuses of no-take protected areas.

## Code availability

The workflow consisting of several R scripts used to produce these datasets can be found in the Zenodo repository together with the data files and supplementary figures. The development of code can be tracked on Github here: https://github.com/jkumagai96/Marine_Habitat_protection. All the data presented in the figures in the manuscript can be reproduced with the available code written and run in R Studio (version 1.3.1073 – “Giant Goldenrod” Linux) with R version 3.6.0. The packages used are managed through *renv* with these associated files are available on Github. Please note that the workflow requires the user to download all the habitat data and the union of exclusive economic zones and country polygons from the data sources listed in Table 2.
