# Supplementary material for: Habitat Protection Indexes - new monitoring measures for the conservation of coastal and marine habitats
Source: Sci Data. 2022 May 12;9:203. doi: 10.1038/s41597-022-01296-4 (PMC9098915; doi:10.1038/s41597-022-01296-4)
Supplement: Supplementary file 1 — Supplementary Information 1 [file 41597_2022_1296_MOESM1_ESM.pdf]

Supplementary Information 1 – GPHPI and LPHPI Figures per Habitat

Title: Habitat Protection Indexes - new monitoring measures for the conservation of threatened marine habitats

Corresponding Author: Joy A. Kumagai (joy.kumagai@senckenberg.de)

Authors: Joy A. Kumagai & Fabio Favoretto, Sara Pruckner, Alex D. Rogers, Lauren V. Weatherdon, Octavio Aburto-Oropeza, Aidin Niamir

Global Proportion of Habitats Protected Index (GPHPI) and Local Proportion of Habitats Protected Index (LPHPI) per Habitat

In this supplementary material, we present the Global Proportion of Habitats Protected Index (GPHPI) and Local Proportion of Habitat Protected Index (PLPHPI) for each habitat considered in the analysis.

For quick reference the GPHPI indicates the amount of habitat within PCAs (protected or conserved areas) divided by the total global area of the same habitat and the LPHPI indicates the amount of habitat within PCAs divided by the area of the habitat within each jurisdiction.

Table of contents:

a. Supplementary Figure 1: GPHPI and LPHPI for cold corals 2
b. Supplementary Figure 2: GPHPI and LPHPI for warm water corals 3
c. Supplementary Figure 3: GPHPI and LPHPI for knolls and seamounts 4
d. Supplementary Figure 4: GPHPI and LPHPI for mangroves 5
e. Supplementary Figure 5: GPHPI and LPHPI for saltmarshes 6
f. Supplementary Figure 6: GPHPI and LPHPI for seagrasses 7

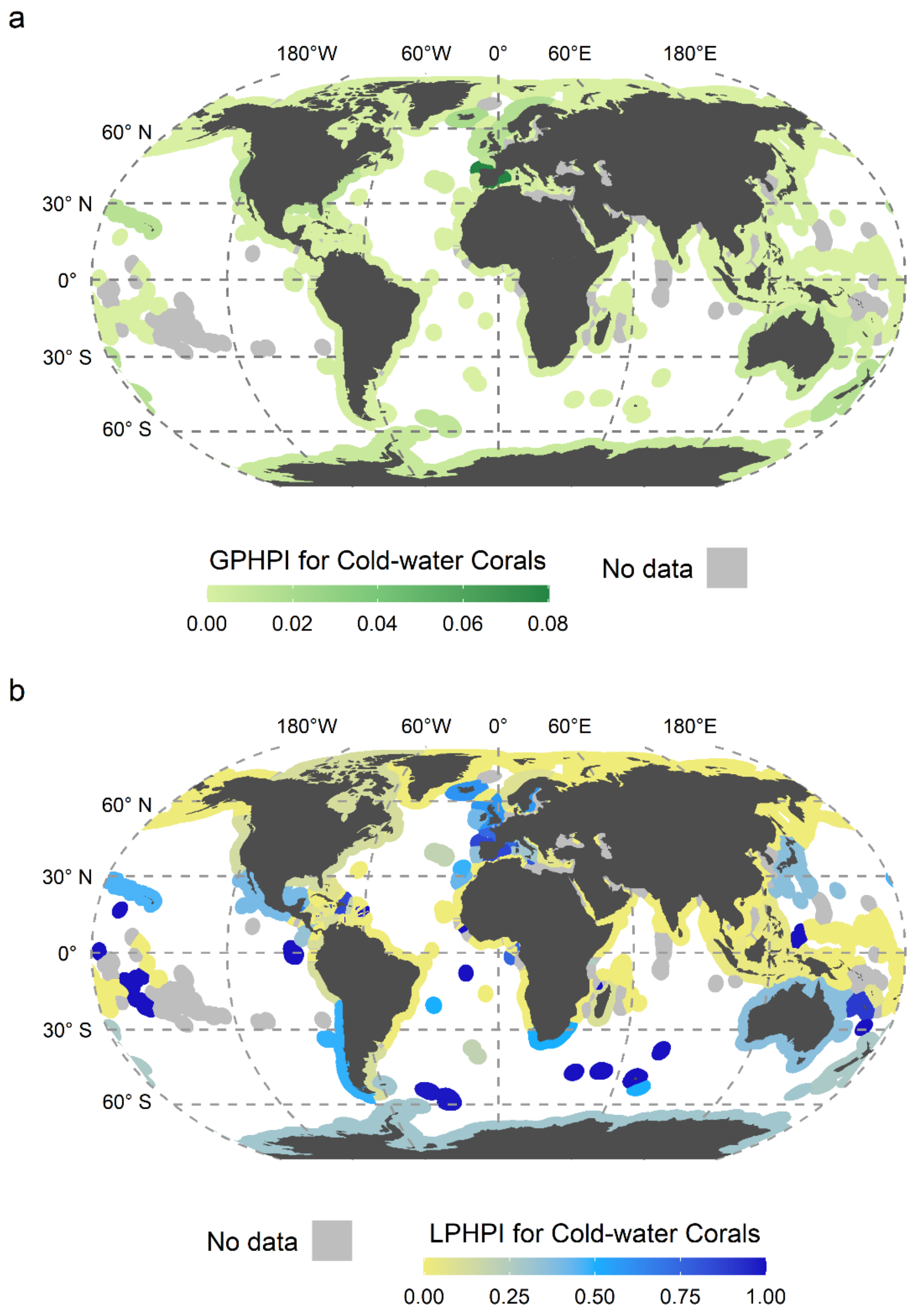

a. **Supplementary Figure 1:** Global proportion of habitat protected index and local proportion of habitat protected index for cold corals. a) GPHPI illustrates the contribution of jurisdictions to the global protection of cold corals, ranging from yellow-green (low contribution) to dark green (high contribution). The index ranges from 0 to 1, but only 0 to ~0.08 is depicted here due to no jurisdictions scoring higher than ~0.08. b) LPHPI illustrates how much a jurisdiction is covering their cold corals with PCAs compared to the maximum habitat extent, ranging from yellow (low contribution) to dark blue (high) contribution.

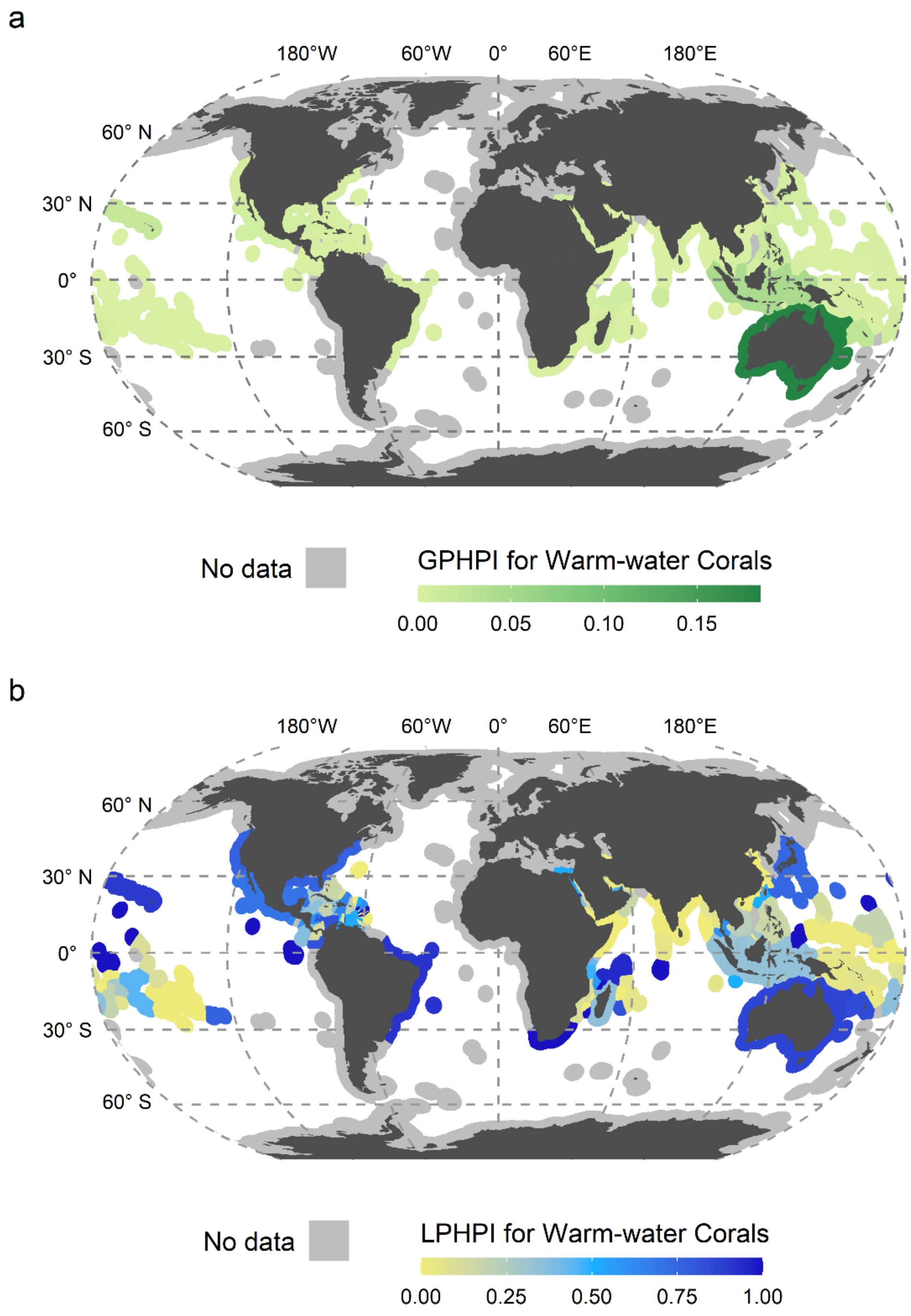

b. **Supplementary Figure 2:** Global proportion of habitat protected index and local proportion of habitat protected index for warm water corals. a) GPHPI illustrates the contribution of jurisdictions to the global protection of warm water corals, ranging from yellow-green (low contribution) to dark green (high contribution). The index ranges from 0 to 1, but only 0 to ~0.18 is depicted here due to no jurisdictions scoring higher than ~0.18. b) LPHPI illustrates how much a jurisdiction is covering their warm water corals with PCAs compared to the maximum habitat extent, ranging from yellow (low contribution) to dark blue (high) contribution.

a

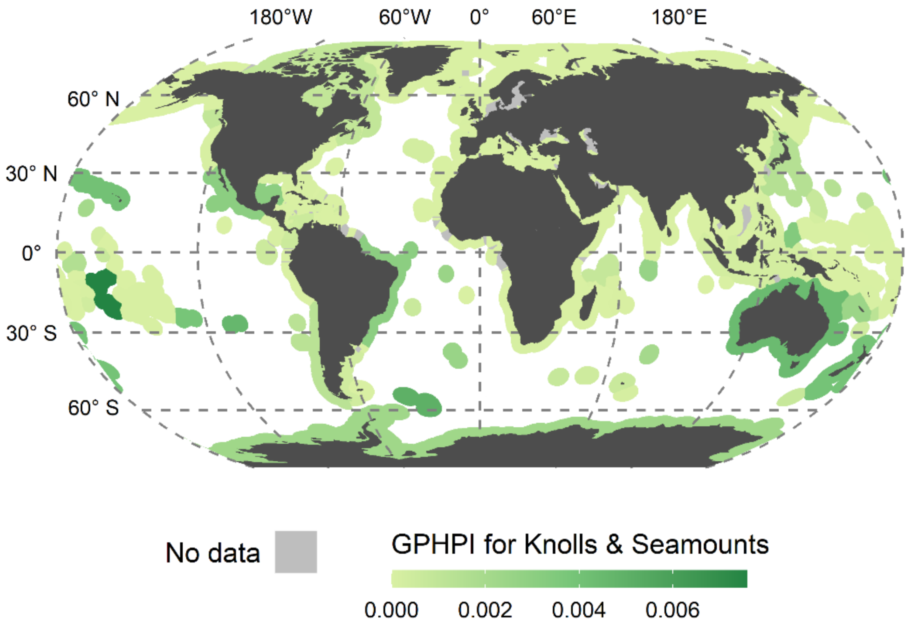

b

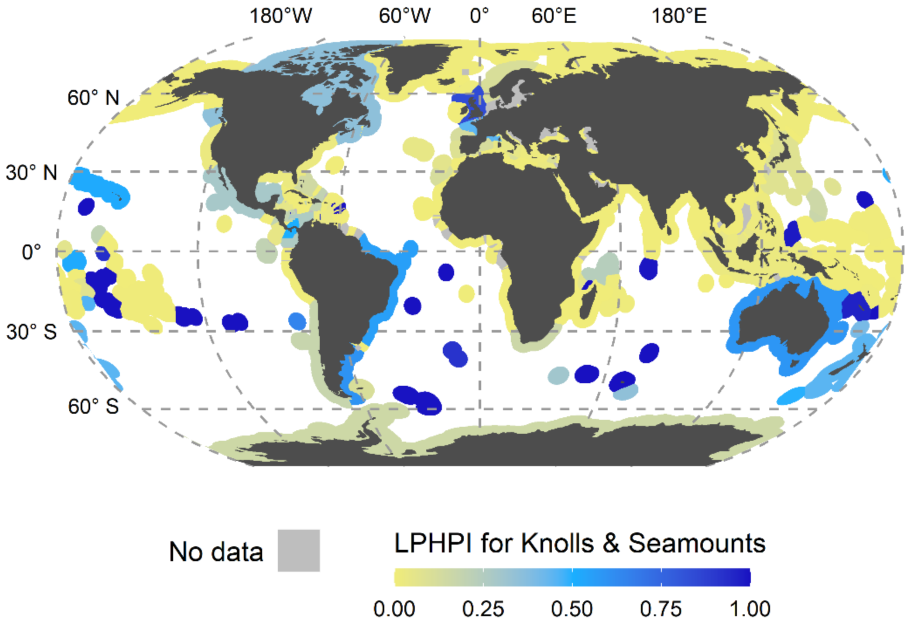

c. **Supplementary Figure 3:** Global proportion of habitat protected index and local proportion of habitat protected index for knolls and seamounts. a) GPHPI illustrates the contribution of jurisdictions to the global protection of knolls and seamounts, ranging from yellow-green (low contribution) to dark green (high contribution). The index ranges from 0 to 1, but only 0 to ~0.008 is depicted here due to no jurisdictions scoring higher than ~0.008. b) LPHPI illustrates how much a jurisdiction is covering their knolls and seamounts with PCAs compared to the maximum habitat extent, ranging from yellow (low contribution) to dark blue (high) contribution.

a

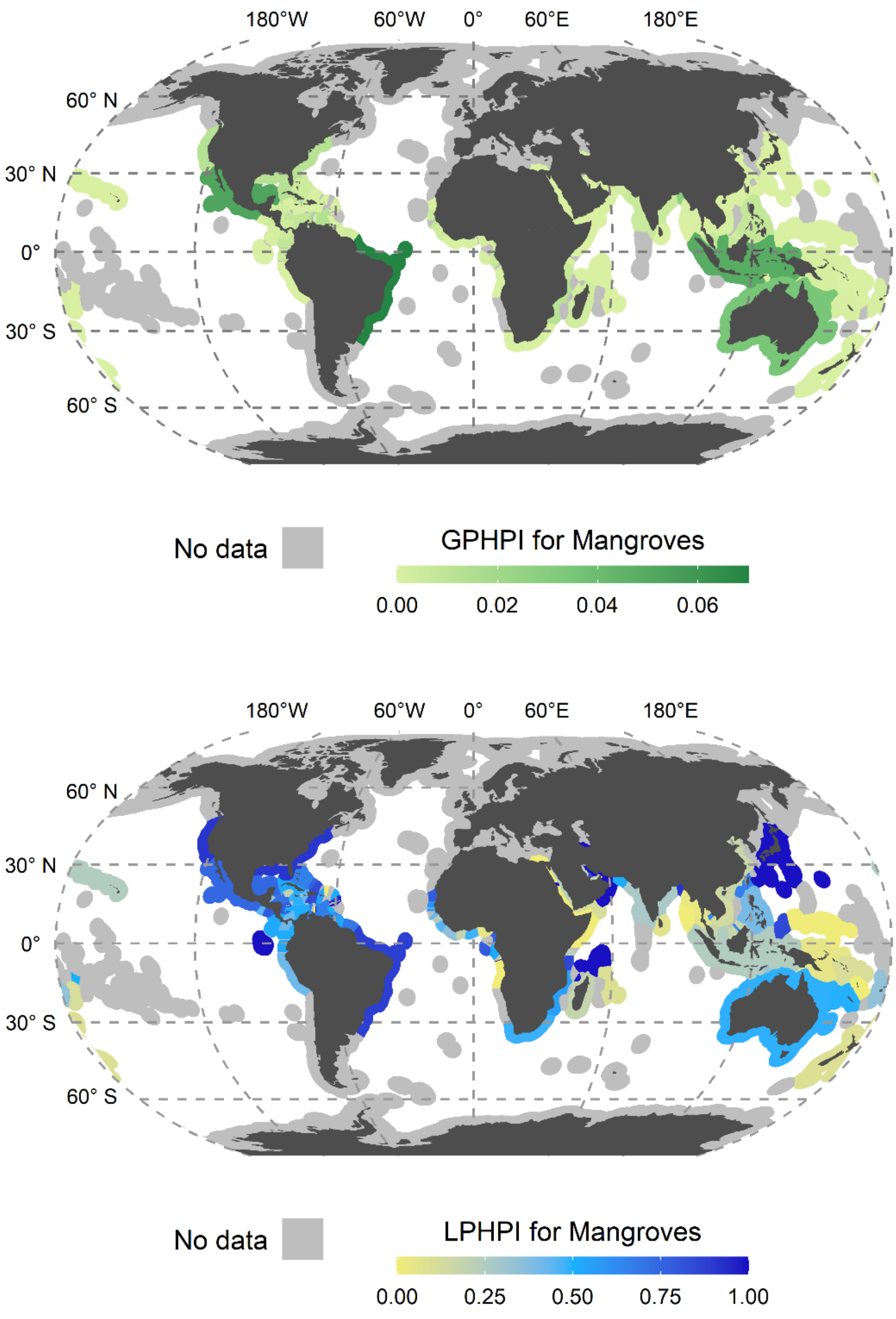

b

d. **Supplementary Figure 4:** Global proportion of habitat protected index and local proportion of habitat protected index for mangroves. a) GPHPI illustrates the contribution of jurisdictions to the global protection of mangroves, ranging from yellow-green (low contribution) to dark green (high contribution). The index ranges from 0 to 1, but only 0 to ~0.07 is depicted here due to no jurisdictions scoring higher than ~0.07. b) LPHPI illustrates how much a jurisdiction is covering their mangroves with PCAs compared to the maximum habitat extent, ranging from yellow (low contribution) to dark blue (high) contribution.

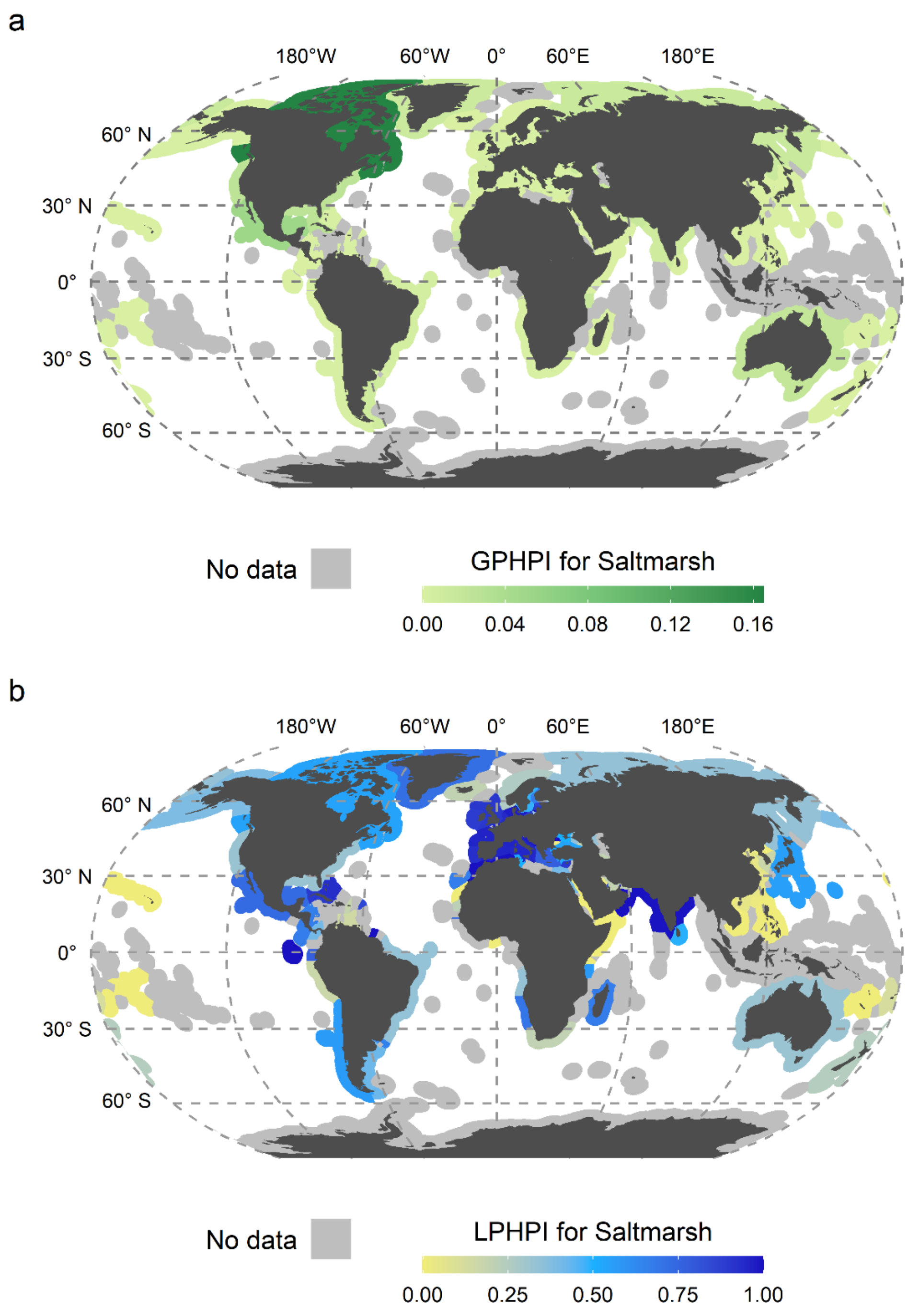

e. **Supplementary Figure 5:** Global proportion of habitat protected index and local proportion of habitat protected index for saltmarshes. a) GPHPI illustrates the contribution of jurisdictions to the global protection of saltmarshes, ranging from yellow-green (low contribution) to dark-green (high contribution). The index ranges from 0 to 1, but only 0 to ~0.16 is depicted here due to no jurisdictions scoring higher than ~0.16. b) LPHPI illustrates how much a jurisdiction is covering their saltmarshes with PCAs compared to the maximum habitat extent, ranging from yellow (low contribution) to dark blue (high) contribution.

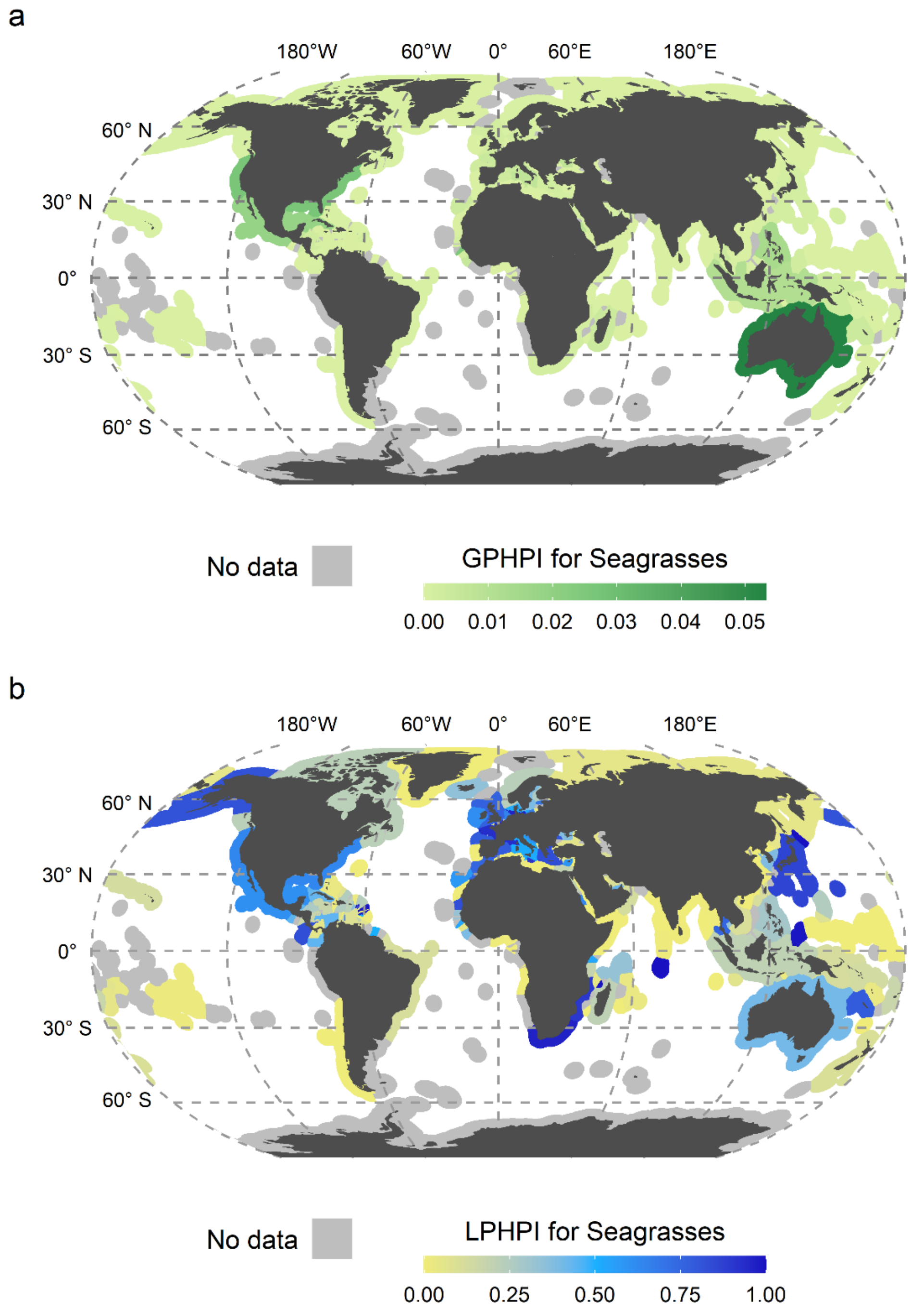

f. **Supplementary Figure 6:** Global proportion of habitat protected index and local proportion of habitat protected index for seagrasses. a) GPHPI illustrates the contribution of jurisdictions to the global protection of seagrasses, ranging from yellow-green (low contribution) to dark green (high contribution). The index ranges from 0 to 1, but only 0 to ~ 0.05 is depicted here due to no jurisdictions scoring higher than ~0.05. b) LPHPI illustrates how much a jurisdiction is covering their seagrasses with PCAs compared to the maximum habitat extent, ranging from yellow (low contribution) to dark blue (high) contribution.
