# Supplementary material for: Habitat Protection Indexes - new monitoring measures for the conservation of coastal and marine habitats
Source: Sci Data. 2022 May 12;9:203. doi: 10.1038/s41597-022-01296-4 (PMC9098915; doi:10.1038/s41597-022-01296-4)
Supplement: Supplementary file 2 — Supplementary Information 2 [file 41597_2022_1296_MOESM2_ESM.pdf]

Supplementary Information 2 – 30% Protection Target Figures per Habitat

Title: Habitat Protection Indexes - new monitoring measures for the conservation of threatened marine habitats

Corresponding Author: Joy A. Kumagai (joy.kumagai@senckenberg.de)

Authors: Joy A. Kumagai & Fabio Favoretto, Sara Pruckner, Alex D. Rogers, Lauren V. Weatherdon, Octavio Aburto-Oropeza, Aidin Niamir

30% Protection Target Figures per Habitat

Table of contents:

a. Supplementary Figure 7: Targeted GPHP for cold corals 2
b. Supplementary Figure 8: Targeted GPHP for warm water corals 3
c. Supplementary Figure 9: Targeted GPHP for knolls and seamounts 4
d. Supplementary Figure 10: Targeted GPHP for mangroves 5
e. Supplementary Figure 11: Targeted GPHP for saltmarshes 6
f. Supplementary Figure 12: Targeted GPHP for seagrasses 7

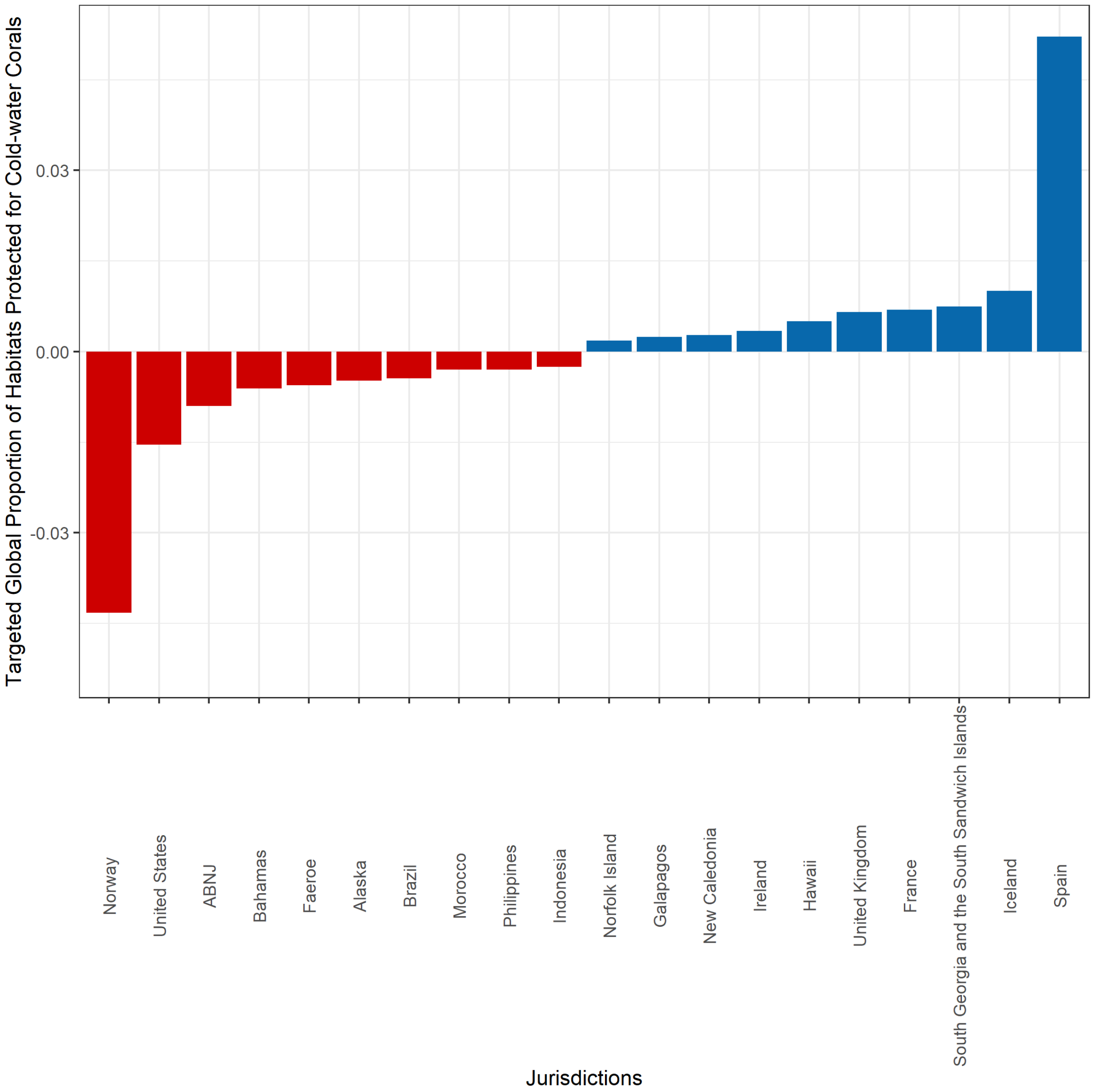

a. **Supplementary Figure 7:** Targeted analysis of the global proportion of habitat protected for cold-water corals. The top 10 and bottom 10 out of 156 jurisdictions ranked according to their targeted global proportion of habitat protected illustrating whether these countries have on average 30% of their cold-water corals within PCAs (protected or conserved areas) and how far away they are from the 30% target. Jurisdictions with a positive value indicate (blue) that more than 30% of their cold-water coral extent fall within PCAs (n = 49), while jurisdictions with a negative value indicate that less than 30% of their cold-water coral extent fall within PCAs (n = 107).

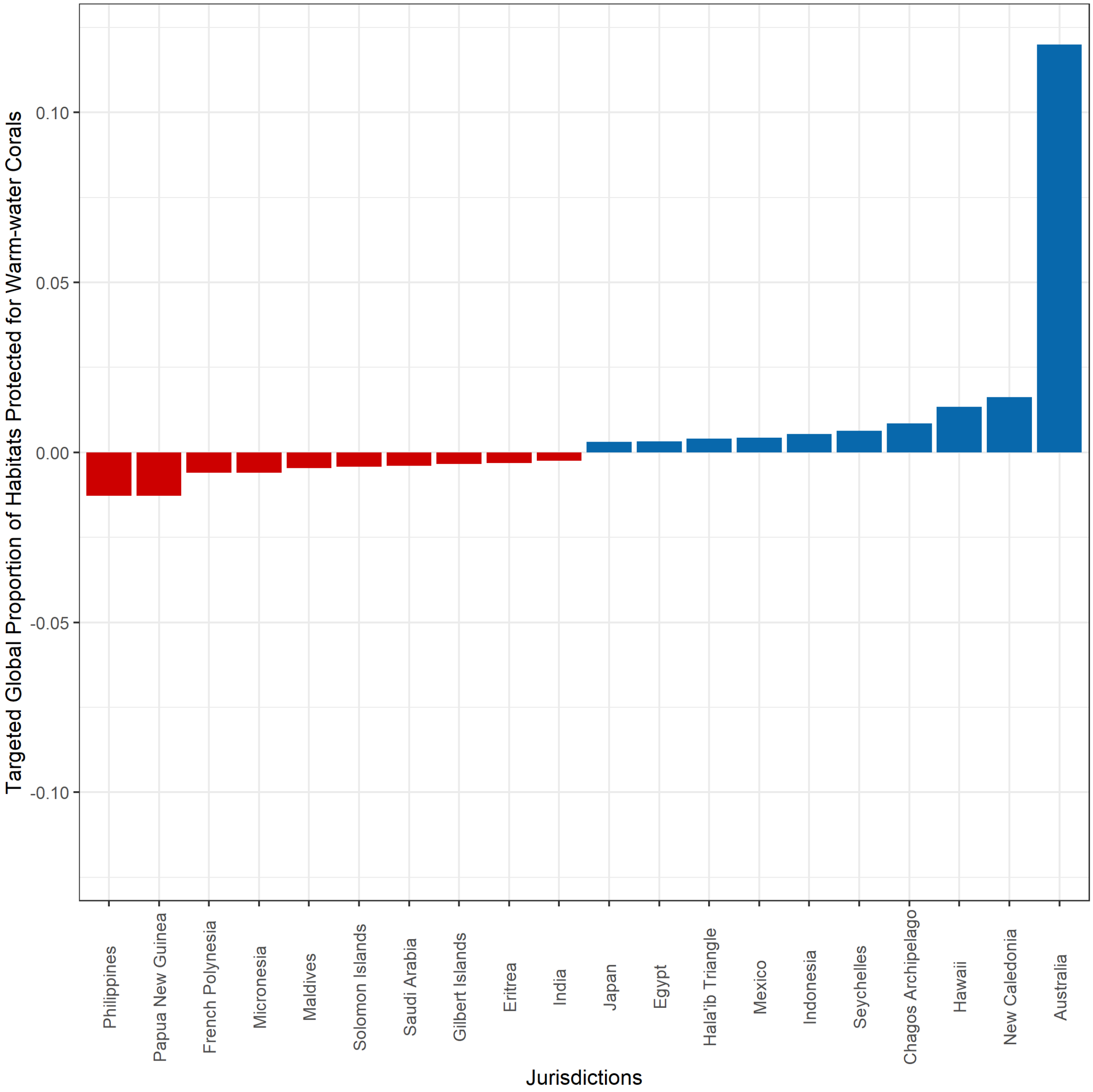

b. **Supplementary Figure 8: :** Targeted analysis of the global proportion of habitat protected for warm-water corals. The top 10 and bottom 10 out of 134 jurisdictions ranked according to their targeted global proportion of habitat protected illustrating whether these countries have on average 30% of their warm-water corals within PCAs and how far away they are from the 30% target. Jurisdictions with a positive value indicate (blue) that more than 30% of their warm-water coral extent fall within PCAs (n = 63), while jurisdictions with a negative value indicate that less than 30% of their warm-water coral extent fall within PCAs (n = 71).

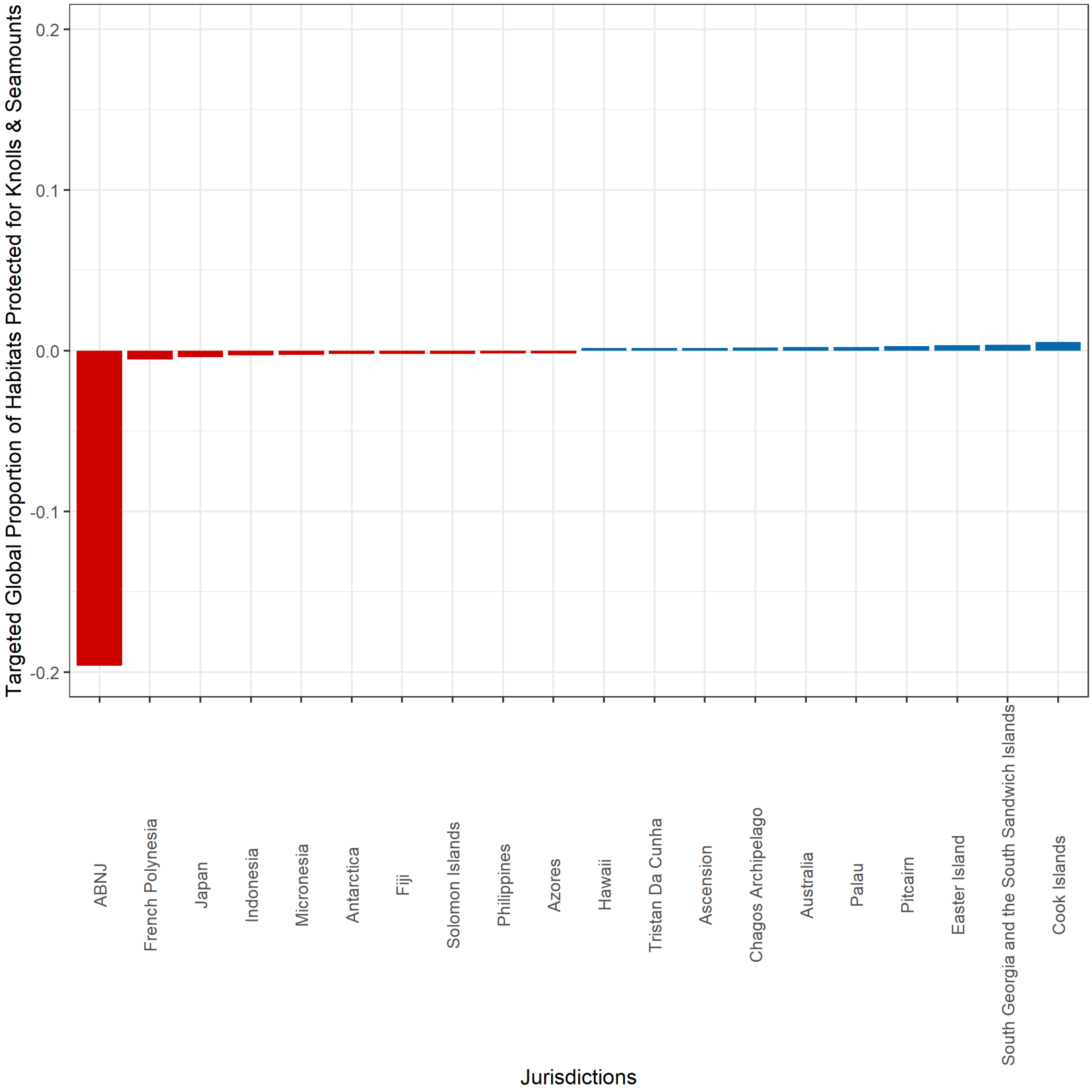

c. **Supplementary Figure 9:** Targeted global proportion of habitat protected for knolls and seamounts. The top 10 and bottom 10 out of 196 jurisdictions ranked according to their targeted global proportion of habitat protected illustrating whether these countries have on average 30% of their knolls and seamounts within PCAs and how far away they are from the 30% target. Jurisdictions with a positive value indicate (blue) that more than 30% of their knoll and seamount extent fall within PCAs (n = 44), while jurisdictions with a negative value indicate that less than 30% of their knoll and seamount extent fall within PCAs (n = 152).

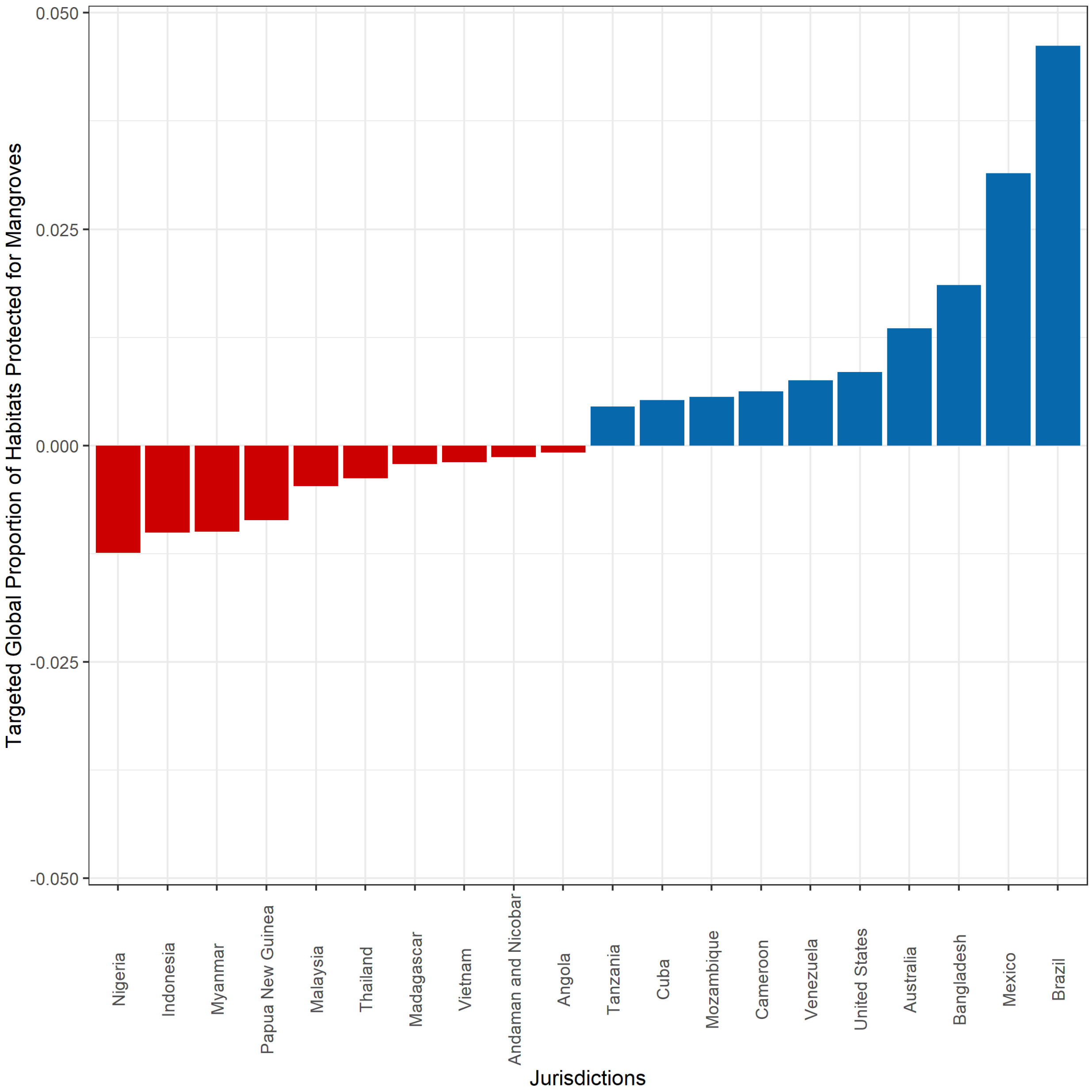

d. **Supplementary Figure 10:** Targeted analysis of the global proportion of habitat protected for mangroves. The top 10 and bottom 10 out of 103 jurisdictions ranked according to their targeted global proportion of habitat protected illustrating whether these countries have on average 30% of their mangroves within PCAs and how far away they are from the 30% target. Jurisdictions with a positive value indicate (blue) that more than 30% of their mangrove extent fall within PCAs (n = 66), while jurisdictions with a negative value indicate that less than 30% of their mangrove extent fall within PCAs (n = 37).

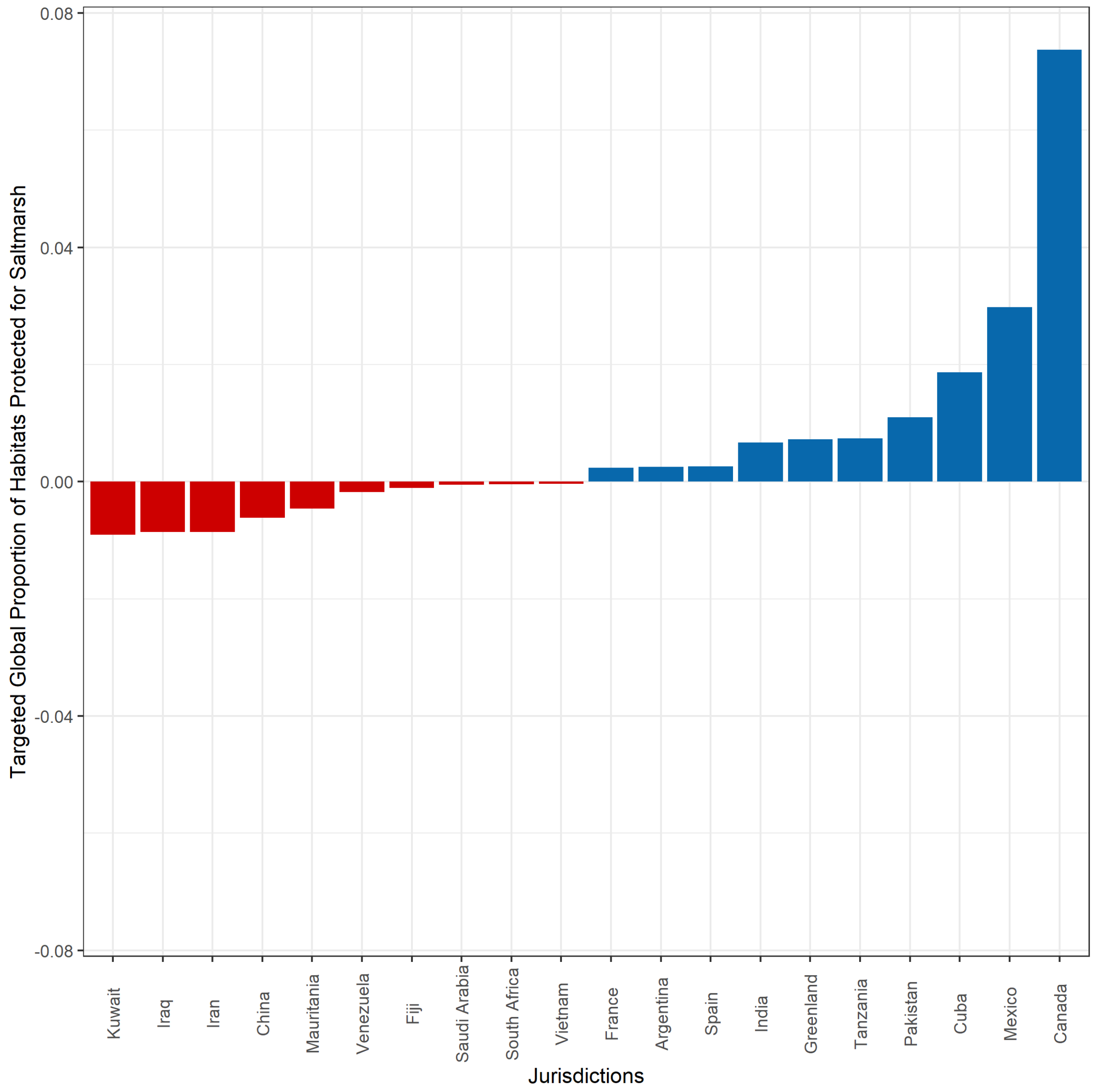

e. **Supplementary Figure 11:** Targeted analysis of the global proportion of habitat protected for saltmarsh. The top 10 and bottom 10 out of 99 jurisdictions ranked according to their targeted global proportion of habitat protected illustrating whether these countries have on average 30% of their saltmarsh within PCAs and how far away they are from the 30% target. Jurisdictions with a positive value indicate (blue) that more than 30% of their saltmarsh extent fall within PCAs (n = 61), while jurisdictions with a negative value indicate that less than 30% of their saltmarsh extent fall within PCAs (n = 38).

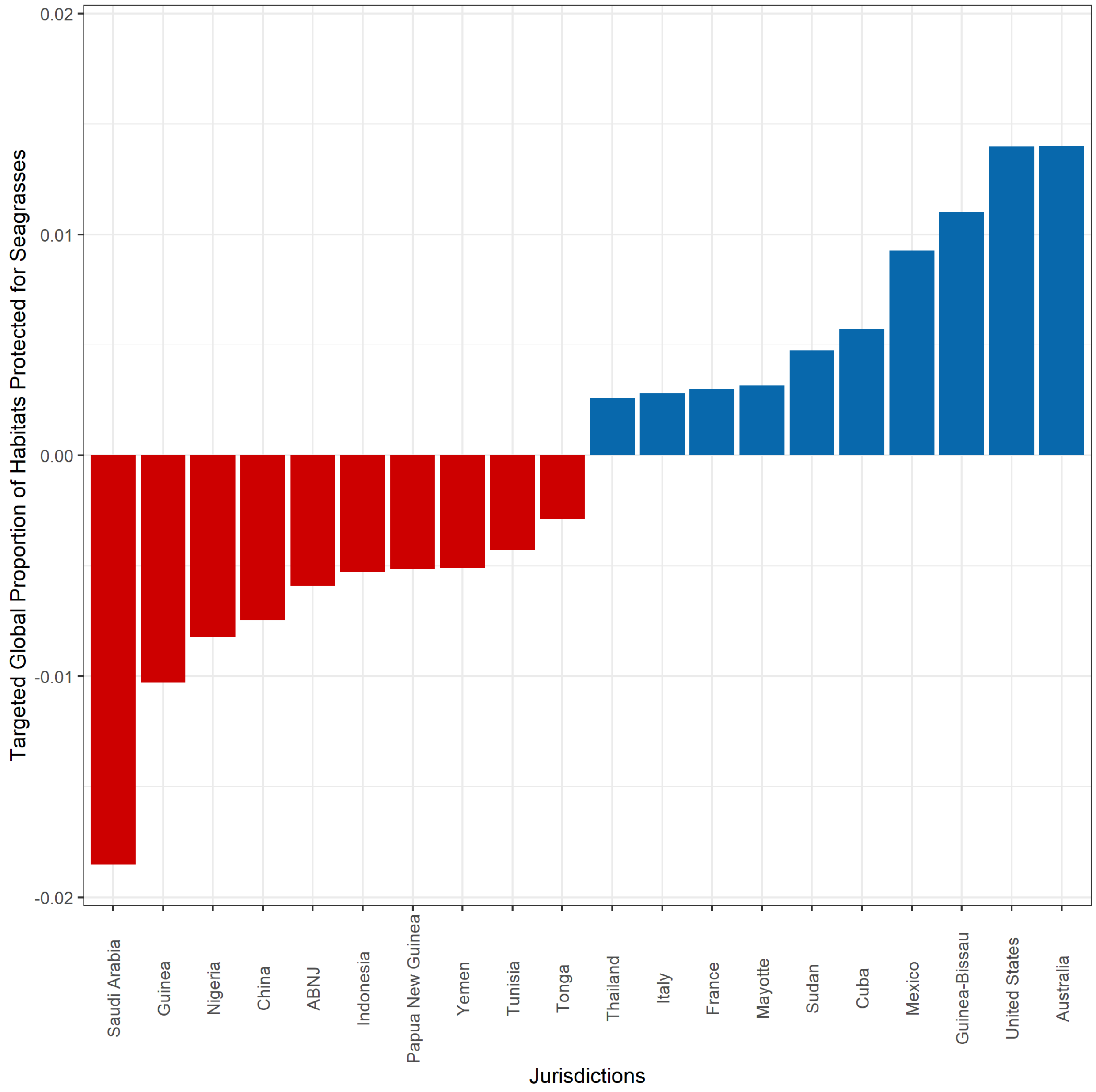

f. **Supplementary Figure 12:** Targeted analysis of the global proportion of habitat protected for seagrasses. The top 10 and bottom 10 out of 165 jurisdictions ranked according to their targeted global proportion of habitat protected illustrating whether these countries have on average 30% of their seagrasses within PCAs and how far away they are from the 30% target. Jurisdictions with a positive value indicate (blue) that more than 30% of their seagrass extent fall within PCAs (n = 61), while jurisdictions with a negative value indicate that less than 30% of their seagrass extent fall within PCAs (n = 104).
